# An Intelligent System Proposal for Improving the Safety and Accessibility of Public Transit by Highway

**DOI:** 10.3390/s150820279

**Published:** 2015-08-18

**Authors:** Carmelo R. García, Alexis Quesada-Arencibia, Teresa Cristóbal, Gabino Padrón, Ricardo Pérez, Francisco Alayón

**Affiliations:** Institute for Cybernetic Science and Technology, University of Las Palmas de Gran Canaria, Campus de Tafira, Las Palmas de Gran Canaria, 35017 Las Palmas, Spain; E-Mails: aquesada@dis.ulpgc.es (A.Q.-A.); teresa.cristobalb@gmail.com (T.C.); gpadron@dis.ulpgc.es (G.P.); rperez@dis.ulpgc.es (R.P.); falayon@dis.ulpgc.es (F.A.)

**Keywords:** ubiquitous computing, ambient intelligence, intelligent transport systems

## Abstract

The development of public transit systems that are accessible and safe for everyone, including people with special needs, is an objective that is justified from the civic and economic points of view. Unfortunately, public transit services are conceived for people who do not have reduced physical or cognitive abilities. In this paper, we present an intelligent public transit system by highway with the goal of facilitating access and improving the safety of public transit for persons with special needs. The system is deployed using components that are commonly available in transport infrastructure, e.g., sensors, mobile communications systems, and positioning systems. In addition, the system can operate in non-urban transport contexts, e.g., isolated rural areas, where the availability of basic infrastructure, such as electricity and communications infrastructures, is not always guaranteed. To construct the system, the principles and techniques of Ubiquitous Computing and Ambient Intelligence have been employed. To illustrate the utility of the system, two cases of services rendered by the system are described: the first case involves a surveillance system to guarantee accessibility at bus stops; the second case involves a route assistant for blind people.

## 1. Introduction

Mobility is a basic necessity for people; as a result, modern societies are concerned with developing transit systems that satisfy this need. Because a critical aspect of transit systems is their accessibility, authorities have developed guidelines, recommendations, and initiatives with the objective of permitting access to transportation for all people, including people with special needs [1,2]. According to the United Nations, the world population in 2011 consisted of seven billion people [3]. The percentage of the population that functions with a disability is approximately 15%, which comprises the largest minority population in the world. Due to the increased life expectancy of the population in economically developed countries, the percentage of the population that live with a disability has increased.

Transit systems provide services with the objective of facilitating access to transit networks; however, these services are designed for people with full physical and cognitive abilities. In this paper, we present the case of an intelligent system for public transit by highway, in which the main objective is to facilitate access to public transit for people with special needs, e.g., the blind, the deaf, people with reduced mobility, or seniors. An important feature of this system is deployment using prevalent components in highway public-transit infrastructure, e.g., sensors, mobile communications systems, and positioning systems. The system is designed to operate in non-urban transport contexts, e.g., isolated rural areas, where the availability of basic infrastructure, such as electricity and communications infrastructures, is not always guaranteed. To construct the system, the principles and techniques of Ubiquitous Computing and Ambient Intelligence have been utilized. The Ubiquitous Computing paradigm has been used for context awareness: to recognize different environments in the transit network and implement smart services. The principles and objectives of Ambient Intelligence have inspired the design and development of services that improve the accessibility of public transit for people with special needs, using sensors and mobile communications.

This paper is structured as follows: in the following section, we present a synthesis of related research. In the third section, we present the objectives that have been established and the technological challenges that are encountered. The system is described in the fourth section. The fifth section is devoted to a case study example. The sixth section assesses the impact of the proposed system. The main conclusions and future works are presented in the seventh section.

## 2. Related Studies

Accessibility to public transit for persons with special needs is a topic that has been addressed by different approaches. These approaches can be grouped into three courses of action: the first course of action analyzes the socioeconomic impacts associated with public-transit accessibility problems for this group of people; the second course of action investigates the factors that affect the accessibility and safety of public transit users; and the third course of action consists of case proposals of systems in which the goal is to facilitate the use of public transit for people with special needs.

The ability to access public-transit services is a right and a fundamental need of people because it provides access to basic services, such as healthcare and education. In addition, it is a component of social cohesion because it facilitates the equality of opportunities and determines the quality of life, given that its general use has a minimal detrimental impact on the environment and reduces traffic accidents, as noted by Duvarci [4]. Raje [5] defines a person who is disadvantaged in the use of public transit as a person who has difficulties or cannot make use of public transit due to psychological, physical, or economic difficulties. Transit authorities have become more sensitive to this issue and have promoted studies, recommendations, and legislation with the objective of developing public transit systems that consider the needs of disadvantaged groups to design systems that are accessible, safe, and equitable. Note the best practices guide from the European Conference of Ministers of Transport [6], which describes how to resolve the typical problems of access in different modes of transportation. Currently, a new line of public transit system planning that is tailored to the needs of users is being developed in parallel with the concept of smart cities. This new approach takes advantage of the deployment of information services and communications infrastructure for citizens in the context of mobility in urban areas to obtain useful information when designing transit routes tailored to the mobility needs of people. A proposed example along this line is given by Frez [7]: the development of a public transit-route planning system for the city of Santiago de Chile that employs a set of cloud information services (Google Maps, OpenStreetMaps, and WAZE).

Different studies have demonstrated that the use of public transit is much safer than private transportation. An example of this type of study is a study sponsored by the United States Department of Transportation [8]. In the case of people with special needs, the factors that influence transportation safety are even greater than the factors that influence the remainder of the population. In the context of transportation, Shaheen [9] classifies the group of people with special needs into five categories: people with visual impairments, people with hearing impairments, people with cognitive impairments in their reflexes, people with cognitive impairments in attention and memory, and people with physical limitations. In this study, the difficulties that each one of these groups displays are described from a medical viewpoint and in terms of the technical aspects to consider when designing vehicles for safer transportation. According to Mitchell [10], the situations that require special treatment for people with special needs with respect to safety and accessibility in public transit by highway are as follows: access to stops, boarding and disembarking from a vehicle, using payment systems, taking a suitable seat, and the resumption of travel of the vehicle. To appropriately resolve these situations, the transport network infrastructure provides special components, such as access ramps in fleet vehicles, cameras on boarding/disembarking doors, and notification systems with informative multimedia displays at stops and station platforms. Jakubauskas [11] describes how different types of impediments embodied by people with special needs can be mitigated via the use of intelligent transportation systems.

Proposed systems with the objective of improving the accessibility and safety of public transit by highway are listed in the bibliography. In the case of blind people, some proposed systems assist the traveler during his or her trip, according to Sánchez [12], Ivanov [13], Baudoin [14]. These proposals are based on the use of positioning systems, such as GPS, and mobile devices, e.g., PDAs and mobile telephones, to communicate with travelers. In the case of people with reduced mobility, Zhou [15] describes a system that is based on Wi-Fi communication, sensors, and microcontrollers and deployed in urban transit infrastructure (vehicles and bus stops) with the objective of facilitating access to urban transportation service. In the case of people with cognitive impairment, Carmein [16] suggests a set of socio-technological services in the context of urban transit, and Luna [17] proposes a route-assistant based on augmented reality and Ubiquitous Computing, which orients the user on his or her trips by displaying information about points of interest. Using a more general approach, García [18,19] proposes a system architecture and an environment in which multimedia services are developed to facilitate the use of public transportation for people with special needs. A precursor to the described system is the Integrated System for Transport Infrastructure surveillance and Monitoring by Electromagnetic Sensing (ISTIMES) project, in which the goal is the deployment of a surveillance and monitoring system for mass transit infrastructure via the use of a distributed system of sensors located at different points in a transportation network [20].

Table 1 shows the different types of special needs user and the types of services that can facilitate their access to public transportation. In contrast to the systems found in the literature, which consist of individual solutions that require multiple hardware resources and specific dedicated software, the system we propose can be developed and deployed systematically. It utilizes common operating principles and resources, and various information services tailored to the different needs of different special needs user types, thus avoiding deployment of various ad-hoc systems for different user types. Another significant feature is that in order to perform its task, the component elements of its architecture are integrated into existing infrastructure, with minimal impact on its components (equipment and communications infrastructure) and running processes. Moreover, they do not interfere with driver operations, thus avoiding distraction, or with the activity of travelers.

**Table 1:** Special needs user groups and required system types.

Special Needs User Type	Required Intelligent Services
Users with limited mobility (require a wheelchair or assistance to walk, cannot use fingers or arms, coordination problems, limited strength).	Systems to assist in boarding and disembarking from the vehicle.
	Alert system in adapted vehicles.
	Traveler information systems with adapted terminals.
	Adapted wireless payment systems.
Users with visual impairment.	Systems to assist in boarding and disembarking from the vehicle.
	Route assistant.
	Adapted wireless payment systems.
Users with hearing impairment.	Mobile device-based information systems based on visual perception.
Users with cognitive impairment.	User-friendly traveler assistant that provides simple and easy to understand information.

## 3. Objectives

As explained in the previous section, the systems and services for people with special needs that are allocated to improving the accessibility and safety of public transit by highway require infrastructure components, such as positioning systems, components for providing information, and mobile communications systems. These components are deployed in different parts of the transit network, such as stops, stations, and vehicles. In the context of urban transit, this deployment can be achieved because urban areas have electricity, mobile communication coverage, and an urban architecture that facilitates this deployment. However, this infrastructure, which is specific to adapted public transit, must be maintained, which implies a greater cost of transportation infrastructure maintenance, especially for the components located at stops and stations due to deterioration from massive use, vandalism and environmental causes.

**Figure 1:**
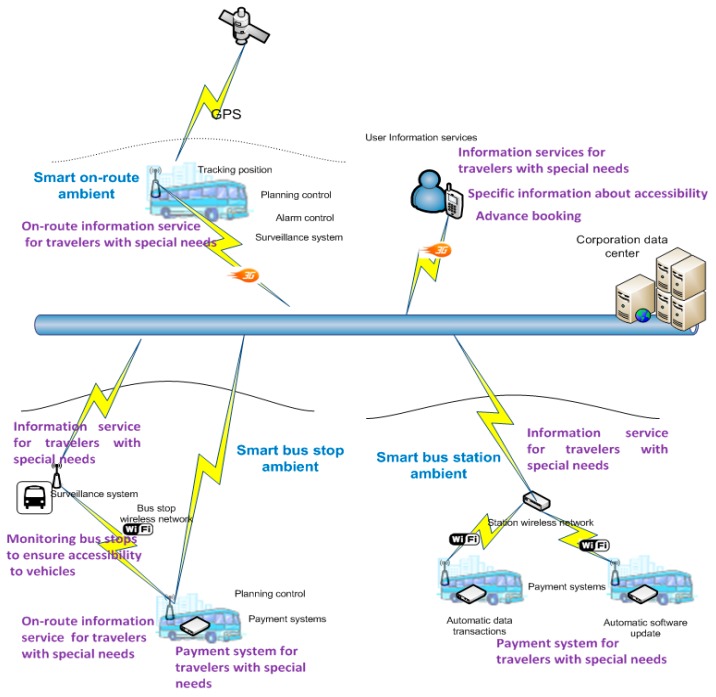
System overview.

The objective of the proposed system is to assist travelers with special needs in the context of highway transportation in urban areas and in isolated rural areas to improve accessibility and safety. In addition, its operation does not imply a significant increase in the maintenance cost of transportation infrastructure. To accomplish these objectives, the system will provide a set of smart services that are deployed in transportation infrastructure. These services are developed by using components that are typically available in transportation infrastructure, as shown in Figure 1. The services provided are autonomous, affecting the normal functioning of the equipment, processes, and services in the transit network as little as possible, that is, the system is transparently integrated into the conventional highway-transit infrastructure. Moreover, the services developed with the proposed system can be deployed alongside traditional support services for public transit travelers, based on audio prompts and information panels. Another aspect is the primary role of vehicles, given that their facilities—both hardware (on-board equipment, sensors, and communication components) and software (positioning system, operation control system, and payment systems)—provide the basic data that are required for the various deployed intelligent services, *i.e.*, a stop-accessibility monitoring system, an adapted payment system, and a route assistant.

## 4. Description of the System

The objective of the proposed system is to facilitate the use of public transit by highway for people with special needs in non-urban areas, such as rural areas. Therefore, the system is capable of operating in geographic locations that lack basic infrastructure, such as electricity and communications. The system utilizes resources that are commonly available in highway public transit infrastructure, in which the resources available in fleet vehicles serve a primary role. Using these resources and applying principles of Ubiquitous Computing, the system provides intelligent environments in different contexts of the transit network (vehicles, stops, and stations) that facilitate access and the safe use of transportation for people with special needs. These smart environments furnish a series of services that naturally and autonomously interact with the user, *i.e.*, they adapt to their needs and transparently operate in the transport infrastructure, affecting existing devices and services in the transportation network as little as possible.

The system is designed to be deployed in the context of intercity public transportation; more specifically, in the key components of a transit network (stations and stops) and in vehicles (see Figure 1). Stations are basic components of the system architecture because they are the points where vehicles, using Wi-Fi communication infrastructure, update their data and on-board applications. These data represent the transit network (routes, stops and time control points) and the vehicle’s planned operations (start of line service and stop schedules), with spatial references (geographic coordinates). The data are used to provide services. In addition, data describing all relevant events that occur during vehicle operation are also downloaded. Since the stops are the physical locations at which passengers embark and disembark, the system considers them to be elements that contain static information (geographic coordinates, lines that pass through the stop, scheduled stop times, equipment and furniture available at the stop, and information on the degree of accessibility for travelers with special needs) and dynamic information (next bus to stop, estimated arrival time and operating bus lines, and issues that affect the use of the stop, *i.e.*, stop out of service or stop moved to another point on the network). With regard to the vehicles, they are a key element of the proposed system because they enable services to be deployed anywhere in the network using their infrastructure. The status of each vehicle is represented at all times by its geographical position, the speed at which it is moving, its present operation (in service, line service, last stop it passed through, next stop, travelers on board and their planned journeys, *etc*.), and the technical condition of its equipment. Using this information and the on-board equipment, the various services are deployed at all the places through which it passes (route information systems, smart payment systems, surveillance systems in vehicles and stops, *etc*.).

### 4.1. Infrastructure Components

Compliance with vehicle arrival/departure schedules, the availability of payment systems that appeal to travelers, and real-time information systems for travelers are features that significantly impact the service quality of highway transport. As a result, transit companies are currently equipped with a series of resources that facilitate real-time control and information for vehicle operations and payment via smart devices (touch-free smart cards and mobile phones). The proposed system takes advantage of the required resources to provide the following functionalities:

- Vehicle positioning system. The positioning system that is most commonly utilized in public transit vehicles is the global positioning system (GPS). This system provides the geographic coordinates of a vehicle (latitude, longitude, and elevation), its measurement quality, and the moment in which it was acquired in coordinated universal time (UTC). The measurements provided by this system may contain errors due to different factors, such as environmental conditions (in the ionosphere and atmosphere), the curvature of the surface of the Earth, shadowed areas that do not enable signals from satellites to be detected, or a random error introduced by the system. A conventional system is assumed to provide measurements with a maximum error of 100 m when the measurement quality is sufficient, which is obtained when the GPS receiver is able to acquire the signal from a minimum of four satellites.
- Communications system. To permit data communications with vehicles, highway public transit companies utilize mobile communication systems, such as General Packet Radio Service (GPRS) or UMTS (3G or 4G) for long-distance communication, Institute of Electrical and Electronics Engineers (IEEE) 802.20 (iBurst) for urban areas, and IEEE 802.11 (WI-FI) for communications at stations. Recently, short-distance communication technologies, such as IEEE 802.15.1 (Bluetooth), IEEE 802.15.4 (ZigBee), or radio-frequency identification (RFID) have been utilized to provide personalized services to people with special needs in different contexts of mobility, such as public transit.
- On-board sensors. To improve vehicle security, vehicles are equipped with open-door sensors. These sensors are used to prevent a vehicle from moving when passengers are entering or exiting the vehicle. In addition, open-door sensors are utilized to activate payment terminals located at the entry and exit points of vehicles. Open-door sensors are connected to the vehicle infrastructure via an electrical connection panel, where the signals from detectors at the end of the carriage assembly of each door are located; from this panel, they are connected to the digital inputs of the payment terminals. When a door is closed, its sensor is in a closed state (normally closed is coded as a logical 0), whereas this state changes to an open state (logical 1) at the moment the door begins to open.

### 4.2. Incorporation of New Components

In addition to the components described in the previous section, which are commonly available in highway public transit infrastructure, the system employs two new components. The following components are added to the vehicle equipment.

#### 4.2.1. On-Board Computer (OBC)

**Figure 2:**
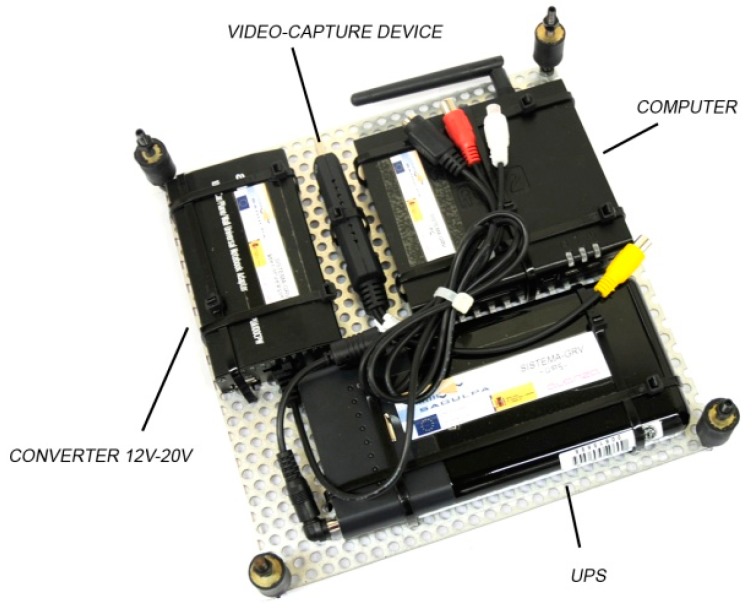
On-board computer and external devices.

This component is a computer with reduced dimensions of 11.5 × 10.1 × 2.7 cm and a weight of 330 grams and a 2 GHz, low-power central processing unit (CPU), 2 GB of double data rate (DDR2) memory, a 64-GB solid-state drive (SSD) drive, four Universal Serial Bus (USB) ports, and a Wi-Fi interface (refer to Figure 2). This computer operates with an electrical power source that can vary between 8 V and 14 V, a maximum consumption of 10 W, and a range of temperatures from 0 °C to 70 °C. In this computer, the processes that provide the data required by the assistants executed on user mobile devices are executed. To provide these data, the on-board computer (OBC) is connected to the vehicle infrastructure and employs the communications system in the vehicle, namely, the Wi-Fi infrastructure.

The OBC runs a lightweight Linux operating system in which the kernel has been adapted to the hardware features of the OBC and required functionalities. The functionalities of the OBC are achieved via a multi-thread system that is composed of the following items:

- The main thread (MTH) that initiates with system start-up and is executed in an uninterrupted manner. This thread creates the remainder of the threads that are executed in the system and the required communication channels.
- Vehicle infrastructure communication thread (CTH). This thread is the first thread created by MTH and executed in an uninterrupted manner. The mission of this thread is to communicate with the vehicle infrastructure to obtain the data required by the services that are provided en route. These data are stored in an area of shared memory to ensure access by the remainder of the threads.
- Service threads (STH). These threads provide various services en route and obtain the data required by the various services; when necessary, they will transmit the data to the mobile devices of travelers.

**Figure 3:**
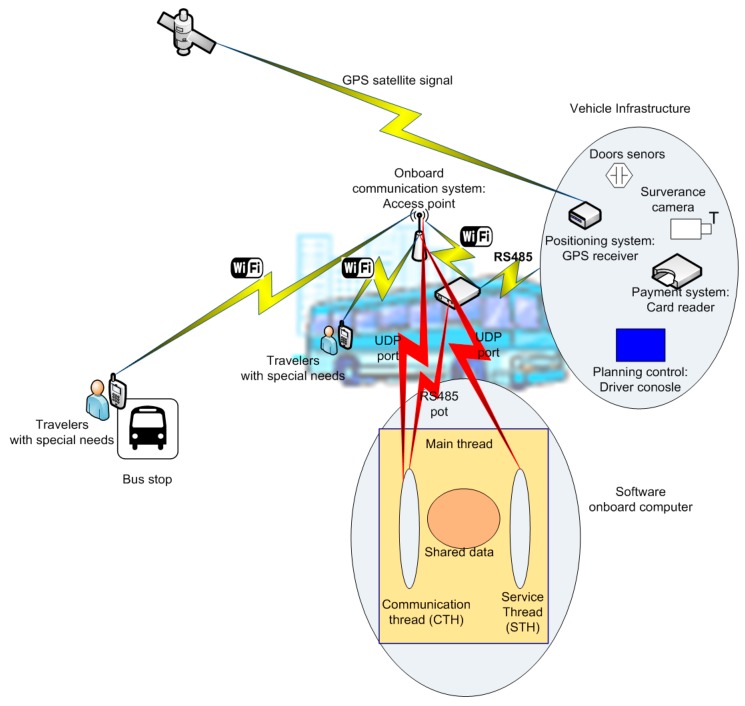
On-board system.

Communication between threads is performed via shared variables. These variables are created by MTH and are inherited by each of its daughter threads. The vehicle operation data are communicated by the vehicle infrastructure, and CTH receives and stores them in these variables. Figure 3 illustrates the different components that operate in the context of the vehicle. The general structure of the utilized data packet is shown in Table 2.

The general operation of a service is explained below. Users board the vehicle at stops, at which point they connect to the services deployed in the vehicle, such as a route assistant or payment service. To do this, they use client applications (ACli applications) installed on their mobile device, *i.e.*, smartphone. Using a service discovery system this application connects to the required service, provided by a service thread (STH) that is run on the on-board computer. Once connection has been established via the communication protocol and the vehicle Wi-Fi infrastructure, all required service data during the traveler’s journey are sent to the ACli application to be processed and displayed to the user. We explain below in more detail how the service threads and the ACli applications function.

The on-board computer (OBC) is the system’s core component, in which every STH thread, providing the various services, is executed. These STH threads read all required data from a set of shared variables that are updated by the CTH thread; this thread is responsible for communicating with the infrastructure element that is responsible for supplying the required data. We explain below in more detail how this communication is conducted via the communication system, and the protocol used. The main element executed on the OBC is the main thread (MTH) which is executed during startup of the OBC operating system. The OBC is connected to the infrastructure via an RS-485 port, and this port is controlled by the CTH thread. The OBC is connected to the mobile user devices via a Wi-Fi interface. To provide services that require sensors that the vehicle infrastructure does not possess, the OBC has different communication interfaces (serial ports, USB, Ethernet, Bluetooth). For example, one of the cases of service developed for this system consists of a bus stop video surveillance system. For this reason a video-capture device has been connected to the OBC, as described below. Finally, in order to provide a silent power supply that is within the voltage range required by the OBC, an electrical power backup unit (UPS) is connected in series between the vehicle battery and the OBC. The UPS isolates the OBC from micro-outages that occur particularly when starting the vehicle.

The key components of the infrastructure providing required data to the proposed system are: the communications system, the positioning system and the vehicle sensors. In the proposed system, the transit company that collaborated in its development, Empresa de Transporte Global Salcai-Utinsa S.A., uses:

- Communications system: UMTS technology (3G) for short data communications relating to operations and alarms control events in the vehicles. Wi-Fi technology for data communications that require higher bandwidth, e.g., to transfer production data generated in the vehicles, synchronize the databases stored on the vehicles or even for software updates run on the vehicle. These data communications are performed using the existing Wi-Fi infrastructure at certain points on the transport network, in stations and garages, where vehicles are frequent and stop for a set period of time.
- Positioning system: GPS system. All vehicles are equipped with a GPS receiver that provides the position, speed and a precise clock signal to synchronize all the clock signals used by the various vehicle devices (driver console and contactless card readers).
- Vehicle sensors: sensors to control vehicle temperature, open-door sensors to determine the state of the vehicle doors (open or closed) and cameras to view the vehicle’s passengers enter and exit.

STH threads follow a standard operating procedure:

Phase 1: Initiation of service. Checking that the connection to the infrastructure is available and that it can provide the data required by the service. This check is performed by accessing the shared data area and verifying the values contained within these data. The CTH thread is responsible for extracting the values from the protocol frames and storing them in the shared variables. If both checks are successful, the service initiates the data.

Phase 2. Announcement of service. In this phase, the service, an STH thread, announces its availability so that the discovery process undertaken by the client applications (ACli) can detect that the service is available. The data required by a service have a variable structure that depends on the service, but all have two common data: the service identifier and a connection address to be used by client applications to connect to the service. A daughter thread, called an Available Services Server (ASS), is responsible for communicating the available service connection data to the ACli applications.

Phase 3. In this phase, the service, an STH thread, waits for connections with the ACli applications running on mobile user devices. For each connection established, the STH thread creates a daughter thread which responds to the ACli application by sending the data required by the service using the protocol. This communication is periodical and the duration of the period depends on the service provided, e.g., a period of five seconds has been established for a “route assistant” service, while for a “payment system” service, this period is 500 ms.

The ACli application also follows a standard operating procedure:

Phase 1. Service discovery. In this phase, the ACli application tries to obtain the connection data from the required service. It does this by trying to establish a connection with the available services announcement system and, more specifically, with the ASS thread. If the connection is successful, the ASS thread sends connection data for the service required by the client application.

Phase 2. Connection to the service. Once the ACli application has the connection address, it tries to establish a UDP connection to the service using the connection address obtained in the previous phase.

Phase 3. Execution of the client application logic. Once connection has been established with the service, an STH thread, the ACli application will periodically receive frames containing the data required by the application. As discussed above, each ACli application has a thread that is responsible for sending these frames.

Considering the different types of user (see Table 1), the information and the way in which it is presented to the user are adapted to their needs. Table 3 shows the resources and requirements of the services (STH threads) and user applications (ACli) for every type of user with special needs.

**Table 2:** Data frame structure.

Field	Field	Field	Field	Field	Field	Field	Field
STX	SRC-ADD	DST-ADD	SEC-NUM	STATUS	DATA-LEN	DATA	CHK

**Table 3:** Resources and requirements for services (STH threads) and user applications (ACli) depending on the special needs of the user.

Special Needs User Type	Resource Infrastructure Required by Services (STH Threads)	Requirements for User Applications (ACli)
Users with limited mobility (require a wheelchair or assistance to walk, cannot use fingers or arms, coordination problems, limited strength...)	Systems to assist in boarding and disembarking from the vehicle. √ Door status: open/closed door sensors and door cameras. √ Vehicle access ramp status: vehicle access ramp deployment sensors. Alert system in adapted vehicles. √ Local wireless communications. √ Location of vehicle: vehicle position. Traveler information systems with adapted terminals. √ Local wireless communications. √ Position and location of vehicle: vehicle position. Adapted payment systems. √ Door status: open/closed door sensors and door cameras. √ Position and location of vehicle.	Dependent on type of mobility limitation and user preferences: √ Textual interaction. √ Video/Graphic interaction. √ Audio interaction. √ Notifications by vibration.
Users with visual impairment	Automatic systems to assist in boarding and disembarking from the vehicle: √ Door status: open/closed door sensors and door cameras. Route systems. √ Local wireless communications. √ Location of vehicle: vehicle position. √ Door status: open/closed door sensors and door cameras. Adapted payment systems. √ Local wireless communications. √ Position and location of vehicle. √ Door status: open/closed door sensors and door cameras.	Smart mobile terminals: √ Audio interaction. √ Notifications by vibration.
Users with hearing impairment	Information systems based on visual perception. √ Local wireless communications. √ Position and location of vehicle. √ Door status: open/closed door sensors and door cameras.	Smart mobile terminals: √ Textual interaction. √ Video/Graphic interaction. √ Notifications by vibration.
Users with cognitive impairment	User-friendly traveler assistant that provides simple and easy to understand information. √ Local wireless communications. √ Position and location of vehicle. √ Door status: open/closed door sensors and door cameras.	Dependent on type of cognitive impairment and user preferences: √ Textual interaction. √ Video/Graphic interaction. √ Audio interaction. √ Notifications by vibration.

#### 4.2.2. Camera

**Figure 4:**
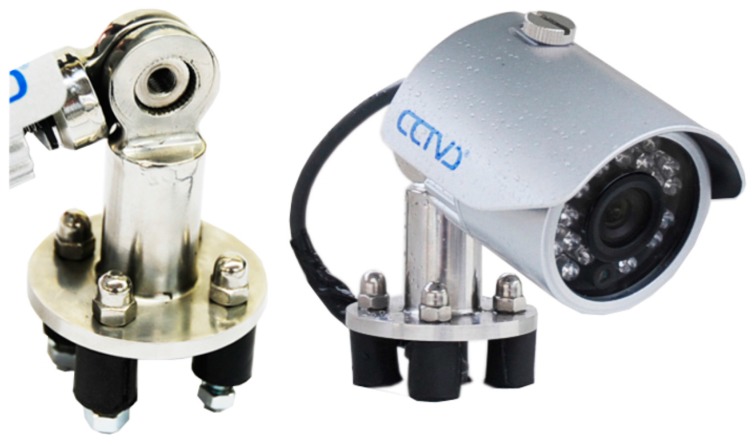
Camera (**right**) and mechanism of attachment to the body of the vehicle (**left**).

This image sensor contains 23 infrared LEDs for night vision with a maximum range of 20 m (refer to Figure 4). The control of gain, brightness, and clarity is automatically achieved. Because it is an external component, it can withstand adverse environmental conditions (dust and water); as a result, it has an IP66 certification. In the developed prototype, installation was performed with the following specifications:

- Location: in the center of the upper front part of the vehicle, at an elevation that varies between 3.2 and 3.7 m, depending on the model of the bus.
- Viewing angle: 45°.
- Focal length: 8 mm.
- Inclination of the camera: 15°–20°.
- Light sensitivity of the sensor: 0.05 lux (night vision).
- Image resolution: 640 × 480 pixels.

#### 4.2.3. Communication System

OBC is integrated into the vehicle infrastructure by RS485 asynchronous communication. RS485 series communication is appropriate for environments that require robustness despite adverse electrical conditions, which may be the case in a public-transit bus. Communication between the OBC and the vehicle infrastructure is achieved by a protocol governed by a component of the vehicle infrastructure—typically the driver console—which assumes the role of master node (MN) of the bus in RS485 communication, with the OBC as the slave node (SN) of this communication. The general format of the data packets exchanged between the nodes is described in Table 2, and the meaning of the fields is shown in Table 4.

**Table 4:** Description of fields from the data frame.

Field	Length	Description
STX	1 byte	Start of O×FF data packet
SRC-ADD	1 byte	Identifier of origin node:
		0: Master node.
		1: OBC.
DST-ADD	1byte	Identifier of destination node:
		0: Master node (MN).
		1: Slave node (SN).
SEC-NUM	1 byte	Packet sequence number: 0–255
STATUS	1 byte	State of infrastructure/vehicle operation:
		0: Infrastructure out of service.
		1: Error in the infrastructure.
		2: Vehicle out of service.
		3: Line in service state.
		4: End of line service.
		5: Forced termination of communication with infrastructure.
		6: Packet correctly received.
		7: Erroneous data packet.
DATA-LEN	1 byte	Number of bytes in the data field.
DATA	Value of the DATA_LEN field	Data field whose structure is dependent on the status (STATUS field).
CHK	1 byte	Byte of checksum for error control in the data packet.

In the context of distributed computing systems, this integration of the proposed system into the existing infrastructure via the communication system is termed a weak-coupling scheme. The advantage of this coupling scheme, as compared to the alternative—Strong coupling—Is that it affects the operation of existing infrastructure elements very little. Specifically, the existing infrastructure will only be affected by the need to incorporate the software module into the MN, which handles the communication protocol.

With the objective of deploying a local wireless network in each vehicle, the communications system has a Wi-Fi access point. The OBC and the mobile devices of travelers that execute the route assistant will be connected to this local network.

The control of the flow of data packets of the protocol is simple. When the MN of the infrastructure sends a data packet, it awaits the response of the SN for a maximum of 200 ms.

- If this length of time passes without a response, then the message is repeated for a maximum number of tries (NT). If the value of NT is attained, the infrastructure assumes a failure in the system and a record and notification of this incident is generated.
- A response is received from the SN; if the STATUS field of the received packet as a value of 6 and the SEC-NUM field coincides with the transmitted packet, the packet has been correctly received by the SN. If the received packet contains a value of 7 in the STATUS field, it indicates that the packet with the number sequence equal to the SEC-NUM field has been received with errors, which causes the MN to resend the packet.

The mobile devices of users are connected to the OBC via Wi-Fi using the User Datagram Protocol (UDP) protocol. The structure of the data packets exchanged between the OBC and a user mobile device coincides with the structure of the previously described packets, with the exception that the packets do not have SRC-ADD and DST-ADD fields because the connection is the UDP. The flow control also matches the previously described flow control. In the event that the connection between the OBC and the mobile device of a passenger is lost, e.g., because the device loses battery power, then the OBC node communicates this situation to the infrastructure with the objective of alerting the driver who can supervise the trip of the passenger with special needs.

The performance of the communication system has been studied in two different scenarios. The first scenario corresponds to services that do not involve mobile user terminals; an example of this type of service is the first use case described below in Section 5.1 (Accessibility surveillance system at stops). The second scenario corresponds to services in which users use their mobile terminals to access the service; an example of this type of service is the second use case described below in Section 5.2 (Route assistant for travelers with special needs). The results of these analyses are presented in each of these sections.

## 5. Use Cases

A system prototype is already available and has been tested in the laboratory. A detailed record of the selected vehicle’s activity during a three-month period was made available for the laboratory tests. The record was possible thanks to the cooperation of the public intercity transportation company, Global Salcai-Utinsa, which operates on the island of Gran Canaria and allowed us access to its infrastructure and vehicles to obtain the required data. This detailed record consists of:

- Record of the vehicle’s GPS position every second (latitude, longitude, elevation, speed, quality of the measurement, when it was acquired).
- Record of vehicle operations and when they were carried out: when service commenced, when the line started operating, stops, travelers’ journey payments, when the line stops, end of service.
- Record of technical alarms associated with the on-board devices and at what time they occurred.
- Record of planning irregularities: failures in line service commencement and stop times, full vehicle warning.

With these data, a simulation was programmed to reproduce a real-life vehicle scenario and the two use cases were tested. The critical aspects that we tested were as follows:

- Correct provision of required data from the infrastructure to the OBC. We checked that the OBC has all the data needed to represent the state of the vehicle at all times.
- Correct provision of data required by each type of ACli application developed. We checked that the ACli client application has all the necessary data to provide accurate and useful information to the user.
- Communication protocol latency between the infrastructure and the OBC. For changes in the state of the vehicle and the OBC, we measured the time elapsed from the moment the change of state occurs at one end (infrastructure or OBC) until it is received by the device at the other end.
- Response times in the interactions between the OBC and the client application. For changes in the state of the vehicle and the client application, we measured the time elapsed from the moment the change of state occurs at one end (OBC or client application) until it is received by the device at the other end.
- Response time of the client application. We measured the time required to display the information to the user according to their preferences.

In the laboratory tests, we also tested system behavior in anomalous situations:

- Failure of any infrastructure component.
- OBC failure.
- Failure of communication with the OBC.

**Figure 5:**
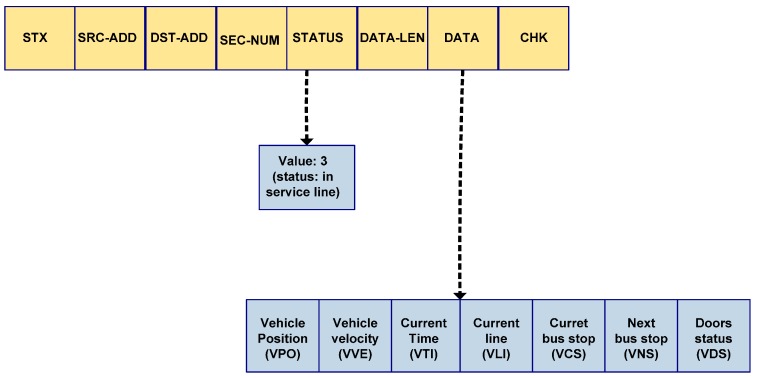
Details of the data communicated via the protocol.

This type of testing is especially important for those services designed for users with special needs: system behavior must at all times be predictable and keep the user informed. To illustrate the utility of the system, two use cases are described. In the first case, the utilization of the system as a tool for supervising accessibility to vehicles at the stops is explained. In the second case, the utilization of the system as a route assistant for the blind is illustrated. In each of these cases, we illustrate the use of the various components and functioning principles explained in the previous section. These systems should function when the vehicle is conducting line service. All data related to the state of the vehicle are communicated by the infrastructure via the protocol described in the previous section. In Figure 5, the structure of the packet utilized for this communication is detailed.

### 5.1. Accessibility Surveillance System at Stops

The accessibility and safety of passengers of vehicles at stops and stations is an important aspect of public transit service, given that it impacts the comfort level of the service rendered, the commercial speed of the vehicles, and the circulation of remaining vehicles. If the area set up for the parking of public transit vehicles is not distinct, the bus must park outside of this area, which implies the following conditions:

- A risk for passengers, given that they must enter the thoroughfare on occasion.
- An inconvenience because they must move outside the vehicle access area, carry luggage, and accompany children or people with mobility problems. Parking outside of the parking area at stops may create access mechanisms for people with mobility problems, e.g., access ramps do not deploy, which impedes access to these travelers.
- Decreased commercial speed given that the vehicle must perform complex maneuvers for parking, which enables passengers to board and disembark.
- Interrupts the circulation of other vehicles, given that they must park by entering the thoroughfare, which increases the risk of accidents.

The surveillance of exclusive public-transit vehicle zones (stops, stations, and bus-only lanes) in urban areas can be conducted using imaging sensor networks installed at main points in the transit network (stops, stations, and in the public right-of-way); an example is the ISTIMES system [20], which has been referenced in the section about related studies. Conversely, this solution is not possible for public transit in non-urban areas because the availability of basic resources (electricity and communications) is not always guaranteed, especially at points in the transit network in isolated rural areas.

The solution proposed by the system consists of utilizing exterior cameras that are installed in vehicles as a type of mobile imaging sensor network. It is a deterrent system for drivers who park their vehicles in areas reserved for public-transit vehicles. When a bus reaches a stop, the system will activate the camera and obtain a series of images of the stop; in the case in which a vehicle is poorly parked, this video will be sent to traffic authorities with the objective of initiating a citation.

The surveillance system is activated when the vehicle is traveling along its route. The required data are obtained from the data frames sent by the infrastructure using the protocol described in the previous section. These data include the vehicle identifier, driver identifier, vehicle operating status, route ID, and vehicle positioning. When the vehicle is performing route service, these data are provided by the infrastructure. Figure 5 shows the packet sent by the infrastructure to the OBC from which these data are obtained. With these data, the system runs as follows:

1. Start of acquisition: Image acquisition is automatically activated when the vehicle is near the next stop on the route. The proximity is expressed by the distance threshold D, which is associated with each stop along the route. Proximity is obtained by calculating the distance between the current position and the geographical point of the stop; when the distance is less than D, the system activates image acquisition. Considering the limitations of measurement accuracy of a conventional GPS, the distance D must not be less than 100 m.
2. End of acquisition: Image acquisition is stopped when the vehicle is more than D from the last stop reached by the vehicle. The acquired images are recorded in a file and stored in the OBC.
3. Referencing images in space and time: To determine where and when the images were acquired, each file is referenced by geography and time (date and time) using data from vehicle positioning.
4. Transfer of image files: When a vehicle makes a scheduled operating stop in a place with Wi-Fi infrastructure (stations, main stops, or garages), the video files are transferred to the image repository of the company.

#### Study of the Performance of the Accessibility Surveillance System at Stops

Prototype tests were performed to analyze four critical features of the system. The first feature is a low resolution image that provides sufficient image quality for the objective. The second feature is the method of image compression, which helps save storage space and bandwidth consumption in transmissions while maintaining sufficient image quality. The third feature is the amount of space required to store the OBC images that are acquired along a route. The fourth is the performance of the communications system. We present the primary results for each of these tests:

- *Resolution*. The resolution must provide sufficient image quality to obtain the required information. In our case, the plate number of the vehicle must be visible. The camera offers several resolutions, which range from 176 × 120 to 720 × 480 pixels. A resolution of 640 × 480 is a suitable resolution for the tests.
- *Method of compression*. Compression reduces the space that is required to store an image sequence and reduce the bandwidth that is required to transmit an image file over the wireless network. Three compression methods were tested: H.260, MPEG-4, and Motion JPEG. We opted for the latter because it is the latest method and offers significant savings in space, namely, compression was performed with 25 frames/s and a resolution of 640 × 480 pixels. With these parameters, the compression of a sequence of images with a length of 1 s requires approximately 34.13 Kbytes of storage space.
- *Storage requirements for a route*. Considering the findings of these tests, the problem consists of estimating the space required for a bus route. According to the previously described system operation, the image sensor is activated when a vehicle is located at a distance of less than or equal to 100 m from the next stop and automatically turns off when the vehicle is located at a distance that is equal to or greater than 100 m from the last stop. Therefore, the acquisition time at a stop is dependent on the time required to travel 200 m around the stop, including the time that the vehicle is stopped for passengers to embark and disembark from the vehicle. To obtain precise information, detailed tracking of a bus has been performed to record the position data of the vehicle (latitude, longitude, elevation, speed, quality of measurement, and instant the measurement was taken) at each second over a long period of time (one month). These records were stored in a database that also contained records of the operations performed by the vehicle. Because these operations records are referenced in time, the time periods during which the vehicle performed programmed trips and the route identification were obtained. After obtaining these periods and noting the geographical coordinates of each route stop, the positioning records obtained during these periods were analyzed by selecting the sets of positioning measurements located within 100 m around each of the stops along the route. For each set, the time instant (T0) when the first positioning measurement was acquired and the time T1 when the last measurement of the set was acquired were obtained, where the estimated space required for each set was (T1 − T0) × 34.13 Kbytes. Table 5 lists the results for the selected route stops. This route has 28 stops in both urban and rural areas. The table lists the identifier of each stop, which coincides with its position in the route order (column 1), the maximum travel time for traversing 200 m around the stop (column 2), and the estimated space required for storing the sequence of images acquired during this maximum time period (column 3). Estimation of the space required to store the image sequence of each stop in the line is 108.75 Mbytes.
- *Performance of the communications system*. In order to assess the performance of the communications system we have only analyzed the time required to transmit the images. We have not analyzed how the mobility of the vehicles affects the transmission error rate because these transmissions are made when the vehicle is stationary. Image transmission is performed when vehicles are in places with Wi-Fi coverage, namely stations and garages. In general, these places are open-plan, *i.e.*, they contain no obstacles that hinder communications. Considering the available bandwidth of the Wi-Fi infrastructure (802.11 g), with speeds in outdoor open environments ranging from 54 Mbit/s in a radius of 75 m to 6 Mbit/s in a radius of 400 m, the estimated transmission times for image files of various sizes are shown in Table 5.

**Table 5:** Results for the study route.

Stop	T. Max (s)	Estimated Maximum Required Storage Space (KBytes)	54 Mbits/seg 75 m (s)	24 Mbits/seg 140 m (s)	6 Mbits/seg 400 m (s)
1	86	2935.18	0.4	1.0	3.8
2	53	1808.89	0.3	0.6	2.4
3	100	3413	0.5	1.1	4.4
4	176	6006.88	0.9	2.0	7.8
5	56	1911.28	0.3	0.6	2.5
6	93	3174.09	0.5	1.0	4.1
7	40	1365.2	0.2	0.4	1.8
8	64	2184.32	0.3	0.7	2.8
9	37	1262.81	0.2	0.4	1.6
10	48	1638.24	0.2	0.5	2.1
11	328	11,194.64	1.6	3.6	14.6
12	214	7303.82	1.1	2.4	9.5
13	251	8566.63	1.2	2.8	11.2
14	409	13,959.17	2.0	4.5	18.2
15	201	6860.13	1.0	2.2	8.9
16	261	8907.93	1.3	2.9	11.6
17	50	1706.5	0.2	0.6	2.2
18	67	2286.71	0.3	0.7	3.0
19	238	8122.94	1.2	2.6	10.6
20	19	648.47	0.1	0.2	0.8
21	46	1569.98	0.2	0.5	2.0
22	14	477.82	0.1	0.2	0.6
23	31	1058.03	0.2	0.3	1.4
24	61	2081.93	0.3	0.7	2.7
25	133	4539.29	0.7	1.5	5.9
26	65	2218.45	0.3	0.7	2.9
27	29	989.77	0.1	0.3	1.3
28	40	1365.2	0.2	0.4	1.8
	TOTAL	109,557.3	15.9	35.7	142.7

### 5.2. Route Assistant for Travelers with Special Needs

This second case of the system is a route assistant for blind people. This assistant uses the data provided by the positioning system and open-door sensors. Using these data, this system improves the accessibility and safety for this type of passenger while traveling on the bus. Next, the principles of the operation of the route assistant are described.

The passenger with special needs, who is a blind person in this case, waits for the bus to arrive at the stop. From the information system of the company, the passenger knows the expected arrival time of the bus that makes the journey required by the passenger, the location of the stop where they must board the vehicle, and the identifier of the line to take.

When the vehicle is preparing to stop, the data connection between the mobile device of the traveler and the OBC of the vehicle is established using the Wi-Fi infrastructure. Once the connection with the OBC is established, a route-assistant service thread (STHi) is created and is dedicated to providing the data to the client application assistant (ACli) that runs on the mobile device of the traveler. At this point, the data packet exchange between the STHi thread and the ACli application begins, using the protocol described in Section 4. Next, the data used by the service, including how they are obtained and their treatment, are described:

- User Profile—This datum indicates the type of traveler that is using the application; in this case, the traveler is a blind person. This datum is stored by the ACli application and sent to the STHi thread.
- Details of the trip the traveler wishes to make, which consists of the line identifier (PLI), the origin stop (POS) and the destination stop (PDS)–These data are transmitted to the STHi thread by the ACli application.
- Current vehicle position—This information consists of the geographical coordinates of the vehicle (VPO), the velocity (VSL) and the time point (VTI) in which the position was acquired. These data are provided by the infrastructure of the vehicle (positioning system), transmitted by the OBC, received by the CTH thread and stored in the shared memory for transmission to the ACli application by the STHi thread.
- Line (VLI)—This datum indicates the programmed bus route provided by the infrastructure of the vehicle (control system operations), which the OBC transmits and the CTH thread receives and stores in the shared memory for the STHi thread to transmit to the ACli application. The infrastructure also provides the number of stops of the route (VNS).
- Current Stop (VCS)—This datum indicates at which stop the vehicle is located if it is paused at a stop or the last stop it passed, if it is not at a standstill. This information is provided by the infrastructure of the vehicle (control system operations), which the OBC transmits and the CTH thread receives and stores in the shared memory for transmission by the STHi thread to the ACli application. The position of the current stop is also provided by the infrastructure (VIS).
- Next stop along the route (VNS)—This datum indicates the next route stop and the estimated time of arrival (VTS). This datum is provided by the infrastructure of the vehicle (control system operations), transmitted by the OBC, received by the CTH thread and stored in the shared memory for transmission to the ACli application by the STHi thread.
- State of vehicle doors (VDS)—This datum indicates whether the passenger access doors of the vehicle are open or closed. This information is provided by the infrastructure of the vehicle (vehicle door sensors), transmitted by the OBC transmits, received by the CTH thread and stored in the shared memory for transmission to the ACli application by the STHi thread.

With these data, the route assistant can help a blind person to safely and comfortably complete a journey. For these travelers, the assistance is rendered by audio prompts, which are played on the mobile device of the traveler. A notable feature of the described assistant related to the data is that the data are based on an ontology inspired by international standards for data models in the field of public transit [21] and Hervas [22], according to Garcia [23]. This ontology defines the concepts, relationships and semantic meaning of the context information, thus enabling interpretation of data in the public transit domain. Conceptually, this domain is divided into four subdomains:

- *The actor domain*. All concepts related to intermodal transit network actors belong to this domain. The main actors are the travelers, transit operators and transit authorities, and the main actor functions are infrastructure, service and infrastructure provider, and service consumer.
- *The infrastructure domain*. Concepts related to basic resources for the implementation of information services in a ubiquitous context belong to this domain (hardware resources, information service providers and client applications). These resources are: computing, communication, location and sensors. This is the most complex domain of the architecture because the concepts and functions envisaged in this area must ensure interoperability of all the information services. Physically, this domain is deployed in the components of the transit network. The transit company is responsible for this domain and the beneficiaries are the transit operators.
- *The service provider domain*. Concepts related to different ubiquitous information services, such as payment systems, traveler assistants, and control operations, belong to this domain. The actors responsible for this domain are transit authorities and operators and the beneficiaries may be operating personnel, transit authority personnel and travelers. Specifications concerning technological and functional aspects belong to this domain.
- *The information consumer domain*. All concepts related to the accessibility, utility and reliability of the ubiquitous client applications belong to this domain. It is therefore a high-level abstraction domain. The actors responsible for this domain are transit authorities and operators and the beneficiaries may be operating personnel, transit authority personnel and travelers.

With regard to the accessibility and safety of a journey using public transit by highway, three critical differentiated phases exist in terms of objectives and data requirements: the boarding phase, the movement phase, and the disembarking phase. In each of these phases, the assistant operates as follows:

1. *Passenger boarding phase*. The objective of the system is safe boarding, which occurs when a passenger boards the correct bus under conditions that do not endanger his or her physical safety that is, boarding when the bus is stopped and the access doors are open. This condition is achieved by indicating to the traveler that the vehicle that they selected is the correct vehicle via the data related to the route (PLI and VLI), current stop (VCS) and destination stop (PDS) and indicating when they can proceed to board, which will occur when the vehicle is stopped, using the vehicle speed data (VSL), and when the doors are open by the vehicle door status data (VDS).
2. *Passenger travel phase*. The objective of the system at this stage is to orient a passenger with special needs to provide safe and stress-free travel. The system communicates the last stop of the vehicle (VCS), the next stop (VNS) if the vehicle is stopped at a stop, the existing stop (VCS and VSL), and the number of remaining stops until his or her destination (VNS and VIS) is reached.
3. *Disembarking phase*. The objective of the system in this phase is safe disembarking. The system achieves this objective by alerting the traveler when he or she has reached his or her destination stop (PDS and VCS), that the vehicle has stopped (VVE), and that the exit doors are open (VDS).

#### Performance of the Route Assistant System

For this type of service, in which users use their mobile phones to access the service, the most influential aspect for system performance is communications. Performance analysis has therefore focused on this aspect. This analysis has consisted in studying the following aspects of the communications system: bandwidth consumed to run the service, degree of mobility supported and battery consumption on mobile user terminals.

- Bandwidth required by the protocol. The protocol feature that most affects the bandwidth consumed is the length of the frames. The protocol establishes a frame structure whose data field is of variable length, the length depending on the current state of the vehicle. The most demanding scenario arises when the vehicle is on a line service, in which case the frames may have a data field with a length of 256 bytes and a total length of 263 bytes. Taking the routes used by the transit company used to evaluate the system as our reference, the maximum number of frames required to represent the state of a vehicle when it is operating a line service is three frames. Considering the available bandwidth of the Wi-Fi infrastructure (802.11 g), with speeds in open outdoor environments ranging from 54 Mbit/s in a radius of 75 m to 6 Mbit/s in a radius of 400 m, these three frames represent a very small percentage of bandwidth.
- Mobility. 802.11 technology was designed to operate in mobile environments in which the mobile stations do not move at speeds above 10 km/h, which is the speed of a person walking. Strictly speaking this is called roaming and not mobility. Because the speed at which a vehicle approaches a stop is variable and depends on various factors, such as traffic or road type, the behavior of the communications system when considering this aspect varies. To obtain precise data on this aspect of performance, we analyzed the behavior of the communications system when the vehicle approaches a stop located at the main station used by the company that collaborated in the development of this system. This station is located in an urban environment with highly variable traffic on the roads depending on the type of day (working or holiday) and the time of day; for example, traffic is heavy on a business day from 07:00 to 22:00. The results show that transmission error rate oscillates between 5% and 10%.
- Battery consumption. The efficient use of energy is a key technology challenge for any mobile communications technology. In our case, considering the currently available technologies on mobile devices—UMTS (3G or 4G), IEEE 802.15 (Bluetooth) and IEEE 802.11 g (Wi-Fi)—Wi-Fi technology was chosen because it has a greater range and bandwidth than Bluetooth. The choice of Wi-Fi over UMTS was due to power consumption. In both technologies one of the aspects that most affects power consumption is the registration of mobile terminals at infrastructure access points, due to fluctuations in the strength of the signal owing to the mobility of the mobile terminal. These registration changes trigger an exchange of control packets from the respective protocols and fluctuations in signal strength when sending the packages. In the topology of the proposed system’s communications network, the Wi-Fi access points are located in the vehicles. Therefore, from the perspective of signal quality and assuming that the user mostly uses the service when aboard a vehicle, this is the ideal situation: the maximum distance between a mobile terminal and the access point will be the length of the vehicle, which in the case of public transit buses is no longer than 20 m, and the vehicle contains no elements (walls) that attenuate the signal. However, in the case of UMTS access, mobile terminals would use various points of infrastructure access (cell registration) that would cause registration changes and fluctuations in the terminal transmission strength, thus causing the mobile terminal to consume more energy. This superior energy consumption behavior of Wi-Fi technology over UMTS will be further accentuated with the incorporation of 802.11AC technology into mobile terminals, which will further improve energy efficiency.

If we take into consideration these parameters for mobile user terminal energy consumption when the user has boarded a vehicle, we can assume that the exchange of the 802.11 g protocol control frames between the terminal and the infrastructure is minimal. It can therefore be disregarded in relation to the frames exchanged between the OBC and the user terminal due to services rendered. The frequency of the transmissions between the mobile terminal and the OBC depends on the service provided. A payment service, for example. The most demanding scenario is a check-in and check-out payment system: the transmissions are effected when the passenger enters (check-in) and leaves (check-out) the vehicle, hence in this scenario, Wi-Fi communications are much reduced. However, in the context of a route assistant, consumption is higher because communications between the mobile user device and the OBC are conducted periodically (every 5 s). This means that on a journey that lasts 60 min at least 1440 frames are exchanged (720 sent by the OBC and 720 responses sent by the client application). If we consider that the frames sent by the OBC have a variable length and that the response of the client application is an 8-byte frame, assuming that all frames sent by the OBC have the maximum size (263 bytes), then the client application receives 184.62 Kbytes and sends 5.62 Kbytes. In the case of the company that has collaborated in the development of this system, the average route length is between 20 min and 150 min.

## 6. Impact Assessment of the System

This section describes how to assess the utility of the system. This assessment will be conducted from two different points of view: that of the public transit user with special needs and that of the transit company.

From the point of view of the transit user, system utility will be assessed according to two parameters. The first is the number and type of users using the system. Users must register for the client application and specify what kind of impairment they have (mobility, sight, hearing or cognitive). This information will give an indication of how many users intend to use the system, the number of potential users and what kind of impairment they have. The second parameter is how many times each user has used the system, what type of service and on what route. These parameters will enable us to objectively assess whether the system facilitates greater use of public transport for these people, as well as discern the type of group that uses it and what type of travel it is used for.

From the point of view of the transit company, the system’s utility is measured by whether it leads to an increase in the number of travelers with special needs using public transportation, and above all, how this use affects planned operations. Indeed, a drawback of people with special needs using public highway transit is that it impacts on the time required to service planned routes. The time required to board and disembark from the vehicle and make a payment is longer than it is for a traveler without special needs. Consequently, vehicles take longer to complete the route. To quantify this increase and whether the proposed system makes it more acceptable, the boarding and disembarking times of passengers at stops will be obtained. These times correspond to the periods during which vehicles are stationary at stops (speed zero indicated by the vehicle’s GPS system). Moreover, as the ACli client application is associated to the type of user that uses it, we will be able to ascertain how this time is affected by user type.

## 7. Conclusions and Future Works

This paper has presented a support system for travelers with special needs in public highway transit. The system improves the accessibility and safety of this group of people when they use this type of public transportation service in urban area and rural areas. The most important aspects of the design of the proposed system include its architecture, which is based on Ubiquitous Computing and Ambient Intelligence, its deployment in different components of the public transport infrastructure, particularly in fleet vehicles, and its use of hardware resources (devices, sensors and communication infrastructure) and services (positioning system, control system planning and traveler information systems) that are usually available in these infrastructures. As a consequence of the re-use of common components of transport infrastructure, the cost of operation and maintenance is significantly reduced.

As a result, the system is able to offer various services that can improve accessibility and safety during the trip of a person with special needs during different phases: boarding a vehicle, en route and disembarking from a vehicle along routes in both urban and rural areas. This ability to provide services in the context of public transit by highway in rural areas distinguishes it from other existing systems because these cases refer to systems that only operate in urban environments.

We conclude that the use of Ubiquitous Computing, Ambient Intelligence and the sensory instrumentation available in public transport infrastructure enables the development of innovative solutions to classic public transportation problems and the development of new services that can improve the use of transportation by and the quality of life of citizens.

For future work we are considering two main lines of action. The first relates to improving the availability of data and services provided by the system. To this end we will incorporate 802.15.4 (ZigBee) technology for communications between the OBC and mobile user devices in order to minimize battery consumption in the user device. We will also look at the possibility of using long-distance mobile communications (3G or 4G) to enable communication between user devices and the operations control center in the event of OBC failure. The second line of action is to develop assistants for people with cognitive impairments, such as the elderly, or mobility impairments. This second area is particularly relevant as it should enable us to respond to certain challenges, such as monitoring passenger safety during boarding and disembarking by controlling the elements involved (access ramps and doors) and, during the journey, by controlling aspects such as wheelchair fasteners and seat belts.
